# Is the Transverse Colon Overlooked? Establishing a Comprehensive Colonoscopy Database from a Multicenter Cluster-Randomized Controlled Trial

**DOI:** 10.3390/diagnostics15050591

**Published:** 2025-02-28

**Authors:** Kristoffer Mazanti Cold, Anishan Vamadevan, Amihai Heen, Andreas Slot Vilmann, Morten Rasmussen, Lars Konge, Morten Bo Søndergaard Svendsen

**Affiliations:** 1 Copenhagen Academy for Medical Education and Simulation (CAMES) Rigshospitalet, Capital Region of Denmark, 2100 Copenhagen, Denmark; kristoffer.mazanti.cold.01@regionh.dk (K.M.C.); anishan.vamadevan@regionh.dk (A.V.); amihai.heen@regionh.dk (A.H.); andreas.slot.cilmann@region.dk (A.S.V.); lars.konge@regionh.dk (L.K.); 2 Faculty of Health Sciences, University of Copenhagen, 2200 Copenhagen, Denmark; 3 Department of Gastrointestinal and Hepatic Diseases, Copenhagen University Hospital—Herlev and Gentofte, 2730 Herlev, Denmark; 4 Danish Colorectal Cancer Screening Database (DCCSD) Steering Committee, 8200 Aarhus, Denmark; morten.rasmussen@reigonh.dk; 5 Bispebjerg University Hospital, 2400 Copenhagen, Denmark; 6 Department of Computer Science, Faculty of Science, University of Copenhagen, 2200 Copenhagen, Denmark

**Keywords:** AI, imaging, assessment, database

## Abstract

**Background and Study Aim:** Colonoscopy holds the highest volume of all endoscopic procedures, allowing for large colonoscopy databases to serve as valuable datasets for quality assurance. We aimed to build a comprehensive colonoscopy database for quality assurance and the training of future AIs. **Materials and Methods:** As part of a cluster-randomized controlled trial, a designated, onsite medical student was used to acquire procedural and patient-specific data, ensuring a high level of data integrity. The following data were thereby collected for all colonoscopies: full colonoscopy vides, colonoscope position (XYZ-coordinates), intraprocedural timestamps, pathological report, endoscopist description, endoscopist planning, and patient-reported discomfort. **Results:** A total of 1447 patients were included from the 1st of February 2022 to the 21st of November 2023; 1191 colonoscopies were registered as completed, 88 were stopped due to inadequate bowel cleansing, and 41 were stopped due to patient discomfort. Of the 1191 completed colonoscopies, 601 contained polypectomies (50.4%), and 590 did not (49.6%). Comparing colonoscopies with polypectomies to those without the withdrawal time (caecum to extubating the scope) was significantly longer for all parts of the colon (*p* values < 0.001), except the transverse colon (*p* value = 0.92). The database was used to train an AI, automatically and objectively evaluating bowel preparation. **Conclusions:** We established the most thorough database in colonoscopy with previously inaccessible information, indicating that the transverse colon differs from the other parts of the colon in terms of withdrawal time for procedures with polypectomies. To further explore these findings and reach the full potential of the database, an AI evaluating bowel preparation was developed. Several research partners have been identified to collaborate in the development of future AIs.

## 1. Introduction

In 2022, 1.9 million cases of colorectal cancer were detected, making it the third most frequent cause of cancer mortality [1]. Colonoscopy remains the gold standard for treating precancerous lesions, and screening programs have proven effective in decreasing mortality [2]. The adenoma detection rate (ADR) is a well established indicator of endoscopist skill level, as it is inversely correlated with post-colonoscopy colorectal cancer [3]. The ADR has a substantial variability, ranging from 8.2–68.1% in the control group of randomized controlled trials [4]. Consequently, these varying endoscopist skill levels result in miss rates of 26% for adenomas, 9% for advanced adenomas, and 27% for serrated polyps [5]. Endoscopists’ performance must be monitored to identify underachieving performances; therefore, several databases have been developed to provide endoscopists with their ADRs as a feedback mechanism for improving performance [6,7]. Establishing the most comprehensive colonoscopy database with trained medical students onsite would ensure the highest level of data integrity, systematically integrating the colonoscopy video recording, pathology findings, and patient-specific outcomes into a structured, invaluable repository of procedural and demographic data [8,9]. In doing so, they offer a powerful resource for clinicians, researchers, and healthcare administrators and provide a training set for artificial intelligence (AI) [10], with several of such datasets with video recordings or images being publicly available [11]. AI has already shown its efficacy in colonoscopy, as both Computer Aided Detection (CAD) and Computer-Aided Quality assurance (CAQ) improve the ADR [12,13,14,15,16]. CAQ has only been tested on a very limited scale [14,15,16], but the CoRS-study (clinicaltrials.gov registration: NCT04862793) was finalized in April 2024 and is the largest randomized controlled trial on CAQ’s impact on ADRs [13]. This study will establish the most thorough colonoscopy database, consisting of video recordings, coordinates of the entire scope movements, patient and endoscopist demographics, and patient and pathological reports.

## 2. Study Aim

To describe a comprehensive colonoscopy database derived from a multicenter randomized controlled trial, exploring its capabilities in terms of identifying procedure-specific quality metrics for the training of future AIs.

## 3. Materials and Methods

### 3.1. Study Design

The study was registered in clinicaltrials.gov (NCT04862793) as a multicenter, cluster-randomized controlled trial, and it received regional ethical approval on the 1st of September 2022 (H-21019106). The study only included patients aged 50–74 as part of the national Danish screening program for colorectal cancer with a fecal immunochemical test (FIT) above 100 μg hemoglobin/liter that were invited to a colonoscopy [17,18]. Patients referred to the three included hospitals in the study (Bispebjerg, Herlev, and North Zealand University Hospital, Hilleroed, Capital Region, Denmark) would receive informed patient consent along with their referral and information letter.

Data collection began for all three sites on 9 February 2022 and ran until 20 April 2024. Designated research assistants were responsible for the data collection and enrollment of the patients.

### 3.2. Training of Research Assistants

Medical students from the University of Copenhagen and the University of Southern Denmark were hired to be research assistants and trained to be responsible for the data collection. They were initially trained in a simulated setting at the Copenhagen Academy for Medical Education and Simulation (CAMES), Rigshospitalet, Copenhagen. The simulated training was conducted by the co-author Anishan Vamadevan, as this is an authorized method for testing a doctor’s competence when conducting colonoscopies in a simulated setting. It consisted of an 8-h training session, where the students were made familiar with the basic concepts of colonoscopy, including anatomical landmarks. Then, they had a one-day observership in a clinical setting where they were trained either by AVa or by a more experienced research assistant to ensure full consistency and uniformity in the data collection. If they encountered any technical issues, they either phoned AVa or the engineer (AH) responsible for the data collection. If the technical issue could not be resolved by phone, AH would correct it the following day.

### 3.3. Data Collection

Patient information: The research assistant registered each patient on a designated Excel sheet which included the following information: name, social security number, sex, age, endoscopist, polyps, cleansing used, and sedation used.

**Figure 1:**
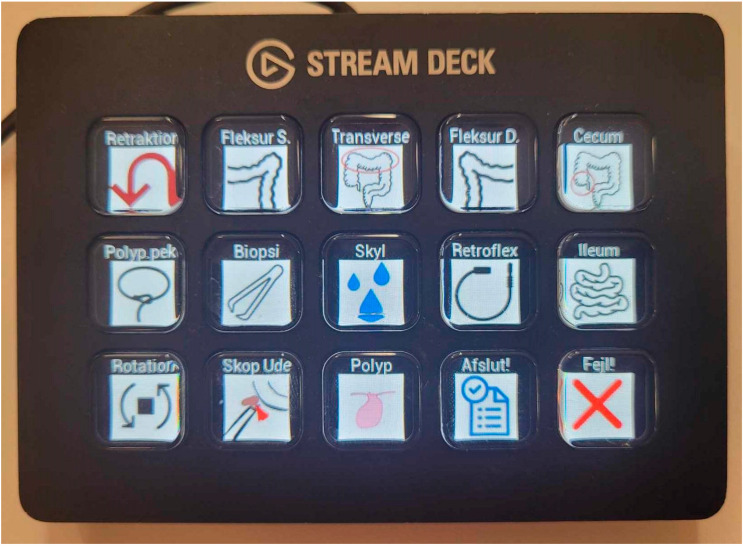
Electronic board (Elgato Stream Deck) used to record the colonoscopy video and events, creating the Logfile (Supplemental Material S1).

**Figure 2:**
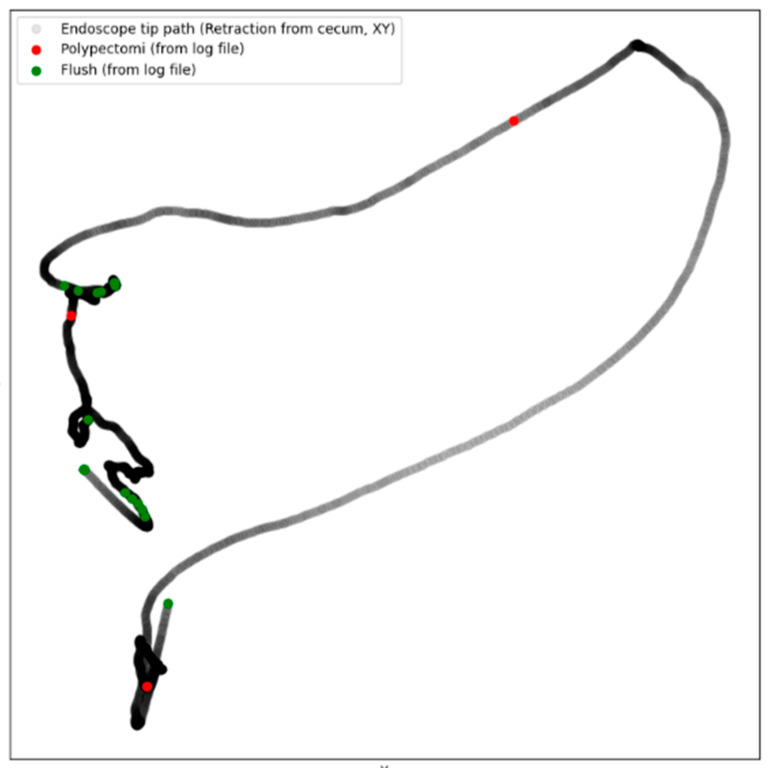
Colonoscope tip track with events from a patient with polypectomies from the left flexure, transverse colon, and sigmoid colon.

- Video and coordinates: When the procedure started, the research assistant would enable the recording of the colonoscopy video along with XYZ-coordinates of the colonoscope from the Olympus ScopeGuide (UPD-3; Olympus Optical, Tokyo, Japan). CAMES has a unique research agreement with Olympus to extract these data through an Olympus receiver box (UCES-3), as described in the following study [19].
- Intraprocedure registration (Logfile): During the procedure, the research assistant would register the following through an electronic board (Figure 1, Elgato Stream Deck, Munich, Germany) by pressing a designated button: position of patient (front, back, left side, right side), anatomical landmarks (right and left flexure, caecum, terminal ileum), withdrawal started, retroflexion, flushing, polypectomy, and polyp spotted. The information was saved as a Logfile with timestamps for the whole procedure (Supplemental Material S1). Combining the Logfile with the coordinates enabled the development of a colonoscope tip and events track (Figure 2).
- Patient-reported discomfort: After the procedure, the research assistant handed the patient a questionnaire about the level of pain and discomfort during the procedure.
- Endoscopist description and pathological report: Four weeks after the procedure, the endoscopist’s description of the procedure was acquired through the local electronic patient journal at the Capital Region of Denmark (Sundhedsplatformen by EPIC systems, Verona, WI, USA) along with the pathological report for each polyp. The epicrisis and complete diagnosis list were also collected.

### 3.4. Data Processing and Establishing Database

All data were saved on a protected cloud service by the Capital Region of Denmark. A script in R (R version 4.1.2, Vienna, Austria) was composed to upload all data into the database established by REDCap electronic data capture tools [20,21]. Through the database, it is possible to export the data and calculate the following: time spent in each colon segment, distribution of polyps, sedation used, patient rotations applied, ADR, Polyp detection rate (PDR) etc. The Colonoscopy Retraction Score (CoRS) [19] and 3D-Colonoscopy Progression Score [22] were calculated using Python and uploaded to the database system as well.

### 3.5. Collaborating Partners

The dataset is included as part of the Intelligent Robotic Endoscopy (IRE) project, financed by the European Union (IRE-101135082-GAP-101135082). Leveraging the data, the project will explore robotic-assisted procedures, ensuring precision, repeatability, and perhaps improved patient comfort. The Department of Computer Science at the University of Copenhagen is the primary applicant for the IRE project, along with other research institutions in Europe. Ambu A/S is a commercial partner of the IRE project and partly funded the establishment of this database.

## 4. Results

A total of 51 research assistants were trained throughout the period, and, as of 21 November 2023, they included 1447 patients with a mean age of 63.2 ± 7.5 years across the three university hospitals. The research assistants spent 737-eight hour shifts on-site, including the patients, collecting the video and scope coordinates, and registering the intraprocedural timestamps (Logfile). They spent 1173 h retrieving the pathological reports and endoscopists’ descriptions of the procedures.

The 1447 colonoscopies were spread across 26 endoscopists from three different university hospitals. A total of 1191 colonoscopies were registered as completed (82.3%), 88 (6.1%) were stopped due to inadequate bowel cleansing, 41 (2.8%) were stopped due to patient discomfort, and, in 127 (8.8%) cases, the Logfile was not generated, with the end-reason of the colonoscopy found to be missing.

Each completed colonoscopy was split into progression (i.e., the time to caecum) and withdrawal (i.e., the time from caecum). Based on the logfile, progression was split into three parts: intubation to left flexure, transverse colon, and right flexure to caecum. Concurrently, the withdrawal was split into three parts: caecum to right flexure, transverse colon, and left flexure to extubating the scope.

Of the 1191 completed colonoscopies, in 601 (50.5%), polypectomies were performed.

The progression time was not statistically significant for any of the three parts of the colon between procedures with and without polypectomies (Table 1); however, the total withdrawal time was longer (mean difference: 496 s, *p* value < 0.001) for caecum to right flexure (103 s, *p* value < 0.001) and left flexure to extubating the scope (375 s, *p* value < 0.001), but not for the transverse colon (13 s, *p* value = 0.92) (Table 1). A total of 121 polypectomies were performed in the transverse colon, with 46 at the right flexure and 32 at the left flexure (the distribution of polyps in the different parts of the colon is presented in Table 2).

For procedures with polypectomies, the patients were not rotated more frequently (mean difference: 0.11 changes, *p* value = 0.06) and the endoscopist did not use retroflexion more frequently (frequency difference: 0.05, *p* value = 0.15). For procedures with polypectomies, the endoscopist used flushing more frequently (mean difference: 2.9 flushes, *p* value < 0.001) (Table 3). Rapifen (Alfentanil) was the only sedative used more for procedures with polypectomies than without polypectomies (mean difference: 70.3 mg, *p* value = 0.029) (Table 3).

## 5. Discussion

This study is the first study to use dedicated on-site research assistants to establish a comprehensive database containing patient information, full colonoscopy videos, scope coordinates, intraprocedural timestamps, patient-reported discomfort, endoscopist descriptions, and pathological reports. In the following sections, we will discuss the preliminary findings and the potential AI development from the database.

### 5.1. Procedure and Segment Durations

A withdrawal time spending a minimum of six minutes evenly distributed across the colon has been recommended to ensure full inspection of the colon wall [23]. This dataset is the first study, to our knowledge, which divides the withdrawal time into the three parts of the colon to monitor even distribution of inspection. Furthermore, we used the scope coordinates to generate a colonoscope tip track of the inspection (Figure 2). The overall withdrawal time for colonoscopies with polypectomies was higher than those without (mean difference: 496 s, *p* value < 0.001), likely due to the time spent performing the polypectomies. However, even though 121 polypectomies were performed in the transverse colon, with 46 at the right flexure and 32 at the left flexure (Table 2), the withdrawal time for the transverse colon was not significantly longer for colonoscopies with polypectomies than without (mean difference: 13 s, *p* value = 0.92). As the transverse colon might be overlooked, another study tried to implement a fixed withdrawal time of at least one minute in the transverse colon, but an increase in the ADR was not detected [24]. As the endoscopist might experience increasing fatigue during the procedure, more thorough inspections of the left part of the colon, with dual inspection studies, may show an increase in the ADR [25]. Unfortunately, no dual inspection studies of the transverse colon exist.

Adenoma miss rates are estimated to be 26% [5], and much emphasis is put on the colonoscopists’ technique, as differences in the ADR have been reported even with the six-minute minimum withdrawal time being met [26,27]. A withdrawal speedometer that alarms the endoscopist when withdrawing too quickly has shown promising results with regard to increasing the ADR [13,16]. The withdrawal speedometer is a promising CAQ system, with several others being developed [28]. This database established a solid pool of data from where future CAQ tools can be developed to enhance the evaluation and assurance of thorough wall inspection by generating a colonoscope tip and events track of the inspection (Figure 2), as withdrawal time does not ensure steady inspection of the mucosa.

### 5.2. Patient Position, Flushing, and Retroflexion

A randomized controlled trial showed that patients randomized in regard to alteration of their position during withdrawal had an increased ADR [29]. Our study did not confirm this finding, as changes in patient position in procedures with polypectomies were not statistically significant compared to procedures without (0.11 changes, *p* value = 0.06). The randomized controlled trial can hold multiple confounders for the increase in the ADR, and, when a patient position alteration is beneficial, it could be explored through AI. As all patient position alterations have a time stamp in this database, it can enable the training of AI to assess the possible increased colonic wall inspection; additionally, if inspection of the different colon parts involves a preferred patient position, it may alarm the endoscopist to alter the position of the patient for a more thorough inspection.

Flushing was used more frequently in procedures with polypectomies than in procedures without polypectomies (2.9 flushes, *p* value < 0.001). It is estimated that 42% of missed polyps are due to inadequate cleansing [30]. The Boston Bowel Preparation Score is the most frequently used preparation score [31]. It relies on endoscopist rating and has shown interrater reliabilities that are fair to strong [32,33]. Tools based on AI have already been applied for bowel preparation, enabling an automatic, instant, and objective evaluation [34]. From this database we have developed an open-source automatic bowel preparation score so that can evaluate bowel preparation and warn the endoscopist when polyps might be missed due to inadequate flushing [35]. We made it open source so it could be further improved to detect other agents blocking the mucosa, such as drug capsules or undigested fragments [35].

Overall, retroflexion did not occur more frequently in colonoscopies with than without polypectomies (0.11, *p* value = 0.15). Retroflexion increased the detection of neoplasia in the distal rectum [36]; however, retroflexion also accounts for 10% of colon perforations [37]. Whether retroflexion should be performed depends on endoscopist competence, the chance of spotting adenomas, and the risk of colon perforation. We encourage other developers and our fellow recipients of the IRE grant to develop AIs that could assess this measure.

### 5.3. Sedation and Patient Discomfort

Rapifen (Alfentanil) was the only one of the sedatives given more frequently in procedures with than without polypectomies (70.3 mg, *p* value = 0.029). This can be a confounder to more skilled endoscopists using Rapifen or another underlying constructs. Patient-experienced discomfort is not reported for this article as it is still part of an ongoing study (NCT04862793), but it will be added to the database. Patient discomfort is an important element of colonoscopies, with most patients experiencing a moderate level of anxiety while undergoing the procedure [38,39], and pain experienced through screening colonoscopies is inversely correlated to the willingness to undergo a second colonoscopy [40]. Reducing patient discomfort has heavily relied on sedative regimes but should also be explored further with regard to endoscopists’ scope manipulation techniques. Providing endoscopists with feedback measures enables a smoother progression [41], and the colonoscopy progression score (CoPS) is correlated with patient discomfort [42]. The presented database allows for using patient discomfort as an outcome for future models.

### 5.4. Limitations and Future Directions in the Training of AI

We established the most thorough colonoscopy database to date; however, our study holds several limitations. For 127 colonoscopies, a Logfile with intraprocedural timestamps was not generated, leaving missing data. The database only included colonoscopies from a FIT-positive screening population in Denmark and could be expanded to include centers in other countries for higher generalizability. The integration of AI technology into colonoscopy holds immense potential [28], relying on access to large datasets for the unique combinations of data for the training of AI algorithms [43]. However, an AI system is only as good as the training dataset used for it and might amplify the bias it is trained on [44]. In this database, the transverse colon might be overlooked, retroflexion and patient positional changes are not associated with increased adenoma detection, and these factors might influence the AIs originating from it. We, therefore, recommend that AI based on this dataset should be tested or trained on other datasets to ensure consistent results [9]. Therefore, we have made our automatic bowel preparation score open source, to ensure external validation, testing, and potential global implementation [35]. AI for other colorectal diseases, such as inflammatory bowel disease, is also an area of immense potential [45,46,47]. Due to the constraints from our ethical approval, we were only allowed to include FIT-positive patients suspected of colorectal cancer, and AIs for other colorectal diseases are therefore not likely be developed from this database. Only 41 (2.8%) colonoscopies were stopped due to patient discomfort, which then received a CT colonography. Much larger databases exist for CT colonography and the training of AI [48,49]. CT colonographies were, therefore, not included in the current study. We did not measure the fatigue level of the endoscopist, which has been shown to decrease ADRs [50].

We did not register time spent performing the polypectomies, which could have divided withdrawal time into polypectomy time and effective withdrawal time. The 121 polypectomies of the transverse colon would lower the effective withdrawal time of the transverse colon, perhaps to an extent where the effective withdrawal time of the transverse colon might be lower for procedures with polypectomies than without, given that the mean difference for withdrawal time of the transverse colon was only 13 s between procedures with and without polypectomies. However, such an assumption is purely speculative as we did not record the polypectomy times, and why the withdrawal time of the transverse colon differs from the other parts of the colon needs to be examined in future studies. Future AIs could address this issue by registering the time spent performing polypectomies. Subtracting the polypectomy time from the total withdrawal time could ensure an effective withdrawal time of above six minutes for all procedures.

Inclusion of full colonoscopy videos provides a rich visual dataset for analyses and training. Scope coordinates offer precise tracking of the endoscope, enabling 3D spatial analytics (Figure 2). Intraprocedural timestamps provide chronological context, aiding in understanding the flow and sequence of the procedure. With the combination of video, coordinates, and clinical reports, there is potential for comprehensive post-procedure reviews and quality assurance. The methodology can be replicated in other medical procedures, expanding the concept of on-site research assistants for data collection. Collaboration with technology and medical device companies could lead to the development of smarter, AI-integrated tools [51]. As none of the AIs developed from this database have been tested yet [35], we have teamed with several research partners to identify further ADR enhancement systems based on AI beyond those already identified [12,13]. We, therefore, encourage high-volume centers to integrate the systematic collection of data in order to establish similar databases like those performed in this study.

## 6. Conclusions

With this study, we present the establishment of the most thorough colonoscopy database, indicating that procedures with polypectomies rather than procedures without polypectomies do not involve a longer withdrawal time in the transverse colon and that neither patient rotation or retroflexion in the rectum does not involve a higher polyp detection rate. The database will serve as the basis of the IRE collaboration, bringing together highly specialized research institutions in AI, computer science, medical education, and private companies. We encourage the development of similar databases for other medical procedures.

## Figures and Tables

**Table 1:** Average time spent in different parts of the colon for procedures with and without polypectomies.

Time Intervals in Seconds	With Polypectomies (*n* = 601)	Without Polypectomies (*n* = 590)	*p* Value
Age, years	63.1 ± 7.59	63.3 ± 7.54	0.26
Male sex	336 (55.9%)	327 (55.4%)	0.71
Total progression	796 ± 478	823 ± 1607	0.70
Intubation to left flexure	359 ± 1542	342 ± 267	0.80
Transverse colon (progression)	251 ± 238	254 ± 235	0.82
Right flexure to caecum	218 ± 268	215 ± 259	0.84
Total withdrawal	1166 ± 518	670 ± 354	<0.001
Caecum to right flexure	339 ± 295	236 ± 199	<0.001
Transverse colon (withdrawal)	322 ± 2052	335 ± 2831	0.92
Left flexure to extubation	603 ± 379	228 ± 204	<0.001

Numbers are displayed in seconds as mean ± sd and compared using an independent samples *t*-test. Displayed as count and frequency and compared using a Chi-square test.

**Table 2:** Distribution of adenomatous polyps from 601 completed colonoscopies with polypectomies.

	Caecum	Ascending Colon	Right Flexure	Transverse Colon	Left Flexure	Descending Colon	Sigmoid Colon	Rectum	Total
Adenocarcinomas	2 (5%)	3 (8%)	3 (8%)	2 (5%)	1 (1%)	1 (5%)	17 (44%)	10 (26%)	39 (100%)
Tubular adenomas	75 (21%)	95 (13%)	34 (5%)	96 (13%)	27 (4%)	47 (6%)	241 (33%)	117 (16%)	732 (100%)
Sessile serrated	25 (21%)	37 (32%)	9 (8%)	23 (20%)	4 (3%)	3 (3%)	13 (11%)	3 (3%)	117 (100%)
Total	102 (11%)	135 (15%)	46 (5%)	121 (14%)	32 (4%)	51 (6%)	271 (31%)	130 (15%)	888 (100%)

Numbers are presented as amount and percent of total.

**Table 3:** Events to increase colonic inspection (retroflexion, patient rotation, and flushing) and sedation used.

	With Polypectomies (*n* = 601)	Without Polypectomies (*n* = 590)	*p* Value
* Retroflexion	406 (0.68)	374 (0.63)	0.15
Patient rotation (count)	0.81 ± 1.1	0.70 ± 1.1	0.06
Flushing (count)	6.4 ± 8.7	3.5 ± 6.0	<0.001
Sedation			
Fentanyl (µg)	76.3 ± 48.9	72.7 ± 43.2	0.24
Midazolam (mg)	2.7 ± 10.1	2.2 ± 7.6	0.41
Propofol (mg)	5.5 ± 44.3	5.1 ± 33.7	0.95
Rapifen (mg)	83.2 ± 241.5	10.2 ± 69.7	0.029

Numbers are displayed in seconds as mean ± sd and compared using an independent samples *t*-test. * Displayed as count and frequency with retroflexion and compared using a Chi-square test.

## Data Availability

The original contributions presented in this study are included in the article/Supplementary Material. Further inquiries can be directed to the corresponding author.

## Supplementary Materials

The following supporting information can be downloaded at: https://www.mdpi.com/article/10.3390/diagnostics15050591/s1, Supplemental Material S1: Logfile registering events performing the procedure according to relative timestamps in seconds and in which part of the colonoscopy recording the event occurs.
