# Supplementary material for: Is the Transverse Colon Overlooked? Establishing a Comprehensive Colonoscopy Database from a Multicenter Cluster-Randomized Controlled Trial
Source: Diagnostics (Basel). 2025 Feb 28;15(5):591. doi: 10.3390/diagnostics15050591 (PMC11898687; doi:10.3390/diagnostics15050591)
Supplement: Supplementary file 1 [file diagnostics-15-00591-s001.zip › diagnostics-3407533-supplementary.pdf]

Supplemental Material S1: Logfile registering events doing the procedure according to relative timestamp in seconds and in which part of the colonoscopy recording the event occurs.

```
Relative_Time;Event;Video;
0;Logging started;I.E.;
Start position is on the left;I.E.;
171;Endoscopy started;I.E.;
221;Flush;vid000.mp4;
405;Fleksur L.;vid003.mp4;
419;Transverse;vid004.mp4;
651;Fleksur D.;vid007.mp4;
824;Cecum;vid010.mp4;
869;Polyp;vid011.mp4;
933;Polypectomi;vid012.mp4;
994;Flush;vid013.mp4;
1041;Flush;vid014.mp4;
1093;Flush;vid015.mp4;
1099;Ileum;vid015.mp4;
1107;Retraction;vid015.mp4;
1111;Cecum;vid015.mp4;
1124;Fleksur D.;vid015.mp4;
1165;Flush;vid016.mp4;
1193;Transverse;vid016.mp4;
1252;Fleksur L.;vid017.mp4;
1311;Polyp;vid018.mp4;
1482;Polypectomi;vid021.mp4;
1626;Flush;vid024.mp4;
2252;Recording ended;vid034.mp4;
2266;End reason - Completed;vid034.mp4;
```
